# Histone-driven hypercoagulation contributes to the lethal triad of acute trauma-induced coagulopathy

**DOI:** 10.1038/s41598-025-12431-7

**Published:** 2025-08-19

**Authors:** Christian Karl Braun, Marco Mannes, Doreen Tabea Spiegelburg, Frederik Müller, Amadeo Klitzing, Anke Schultze, Andreas Bauer, Gerhard Achatz, Markus Huber-Lang

**Affiliations:** 1 Institute for Clinical and Experimental Trauma-Immunology, Ulm University Medical Center, Ulm, Germany; 2 Trauma Surgery Research Group, Department of Trauma Surgery and Orthopaedics, Reconstructive and Septic Surgery, Sportstraumatology, German Armed Forces Hospital, Ulm, Germany

**Keywords:** TIC, Trauma, Platelets, Hemostasis, Coagulopathy, Translational research, Experimental models of disease, Coagulation system

## Abstract

Severe tissue injury and hemorrhagic shock can result in trauma-induced coagulopathy (TIC), acidosis, and hypothermia, denoted as lethal triad. This condition exacerbates trauma complications and contributes to organ dysfunction and mortality, prompting the need to better define its complex pathomechanisms in the blood. Therefore, we developed a standardized ex vivo human whole blood (hWB) model to differentiate the impact of various pathophysiological conditions, damage-associated molecular patterns (DAMPs), and key inflammatory mediators on TIC development. Modelling incremental grades of the lethal triad, we assessed the resulting thrombo-coagulopathy by monitoring platelet activation, clot formation, and mediator release. Both acidosis and hypothermia independently triggered platelet activation (with enhanced surface CD62P/CD63 expression) and platelet consumption, leading to ex vivo clot formation. Completing the lethal triad with additional dilutional coagulopathy revealed even more pronounced effects. Further dissecting underlying cross-talk mechanisms, platelet-rich plasma (PRP) or isolated platelets exposed to similar conditions exhibited no relevant impact on platelet function. Notably, histones—unlike HMGB1, pro-inflammatory cytokines or anaphylatoxins—amplified the lethal triad-driven hypercoagulative TIC response in both hWB and PRP. In conclusion, our findings in a modular, clinically relevant whole blood model highlight histones as a central driver of TIC. Furthermore, tracking early TIC progression in this model provides an effective and efficient platform for evaluating novel therapeutic approaches to target drivers of coagulopathy.

**Supplementary Information:**

The online version contains supplementary material available at 10.1038/s41598-025-12431-7.

## Introduction

Severe tissue trauma and hemorrhagic shock caused by civil, terrorist, and military incidents are the top causes of death among young adults^1^. These events trigger a cascade of physiological responses, culminating in trauma-induced coagulopathy (TIC), a condition characterized by tissue damage, extensive blood loss with massive consumption, maladaptation and reduced generation of clotting factors, platelet dysfunction, activation of innate immunity, and endothelial dysfunction^2^. Metabolic acidosis further exacerbates coagulation problems by altering the activity of proteases involved in both fluid-phase and cellular coagulation^3^. In addition, acidosis compromises multiple clotting functions by platelet consumption, thrombin production, and fibrin polymerization^2^. Post-trauma hypothermia slows biological processes, impairing the function of cascade systems and repair mechanisms^3,4^. In consequence, hypothermia after severe trauma still represents an independent risk factor for mortality^5^. The so called “lethal triad” of coagulopathy, acidosis and hypothermia remains a challenging clinical and scientific challenge and especially a dangerous threat to the patient.

The lethal triad, however, is driven by tissue damage and hypoperfusion^6^ with release of various damage-associated molecular patterns (DAMPs) likewise from Pandora’s box^7^, tissue hypoxia caused by vascular damage and/or hemorrhagic shock^8^, as well as iatrogenic- and auto-dilution during the resuscitation period^9^. While tissue damage and shock synergistically contribute to TIC^10^, further analysis is needed to elucidate the underlying pathways and their respective impacts using clinically relevant models.

Vascular damage, impaired perfusion, and subsequent thrombus formation have previously been investigated in murine models^4^. Furthermore, acute TIC has been modelled in small and large animals, albeit with some limitations^11^. Translational approaches involving in vitro and in silico models aim to delineate the microenvironmental conditions contributing to the lethal triad^12^, with only a few models simulating these conditions using human whole blood^13,14^.

Acute TIC typically develops within the first hours after injury as a hypocoagulable state, while late TIC is associated with hypercoagulability^2^. However, TIC is a highly dynamic entity^15^ that not only aggravates trauma complications^16^ but also perpetuates tissue injury in a “vicious circle”^7^. Despite the well-known clinical consequences of the lethal triad, debates persist regarding its impact on treatment strategies such as damage control surgery^17^. Furthermore, the lethal triad related pathomechanisms, both systemic and within the trauma-induced micromilieu, remain unclear.

In the present study, we aim to elucidate the substantial contributions of both, DAMPs and major pathophysiological conditions to the lethal triad using a standardized ex vivo human whole blood model to define novel mechanistic insights and future research targets.

## Methods

### Donor blood samples

The study was approved by the independent ethics committee of the University of Ulm (No. 103/20) and was carried out in accordance with the Declaration of Helsinki and current local ethical guidelines. After informed written consent, blood was drawn from healthy donors (both male and female) by venous puncture with multifly needles (Sarstedt, Germany) and collected in neutral monovettes (Cat-No.: 02.1726.021; Sarstedt, Germany), preloaded with fondaparinux (FPX; Arixtra^®^; Aspen Pharma Trading Limited, Ireland) for a final concentration of 8 µg/ml, as described before^18^. The tubes were then inverted carefully, and the blood was immediately processed for respective experiments.

### Lethal triad models in whole blood and PRP

**Fig. 1:**
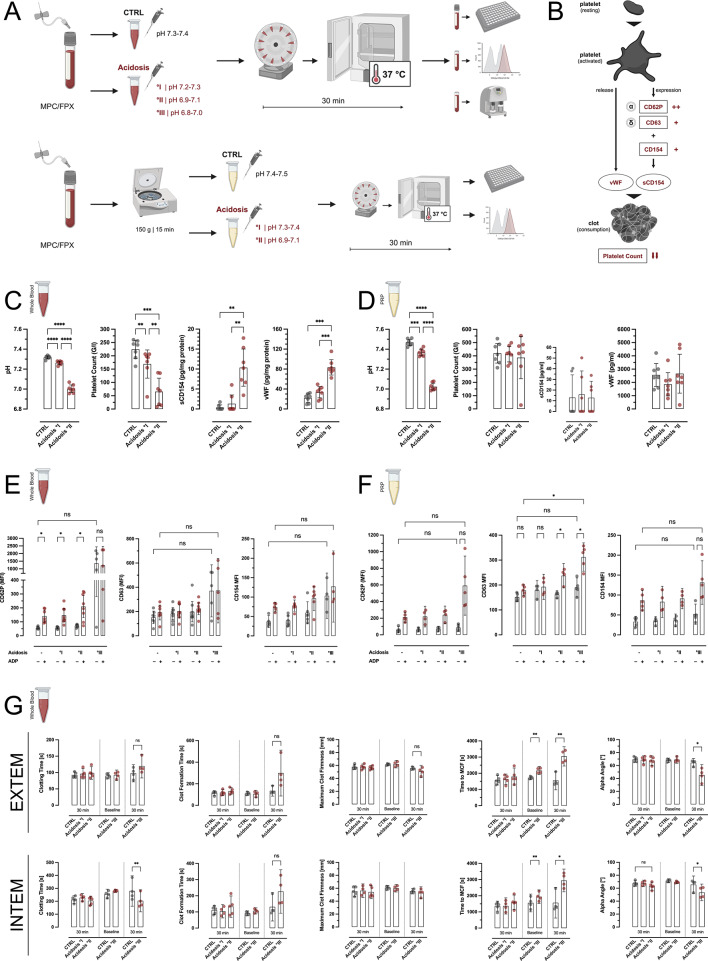
Coagulopathic effects of different grades of acidosis on platelets in whole blood and PRP. (**A**) Protocol for modelling acidosis in whole blood (WB) and platelet rich plasma (PRP). (**B**) Analyzed read-out parameters for platelet activation ex-vivo. CD62P is marker for alpha granule exocytosis, CD63 denotes dense (δ) granule secretion. Surface CD154 (CD40L) is released as truncated sCD154 upon platelet activation. (**C**) Acidosis in ex-vivo whole blood leads to a significant platelet activation, as evident by dropping platelet counts and increasing plasma concentrations of sCD154 and von-Willebrandt factor (vWF), whereas acidosis had no such effect in PRP (**D**). (**E**) Flow cytometry analyses of CD62P surface expression on platelets after 30 min incubation of acidotic WB showed no relevant pre-activation and sustained reaction capacity to post-incubation stimulation with 5 µM ADP at low- and midgrade acidosis (°I/II), but marked activation und loss of reaction capacity after °III acidosis. CD63 expression, which is not inducible by 5 µM of ADP, was slightly increased after °III acidosis, as was CD154 expression. (**F**) Platelets from acidotic PRP showed no pre-activation in terms of CD62P/CD63/CD154 expression, but a slightly enhanced, grade-dependent reaction to subsequent ADP (5 µM) stimulation. (**G**) ROTEM analyses showed no relevant alterations of intrinsic or extrinsic hemostasis at low-/mid-grade acidosis but revealed significant impairment of clot formation and stability under severe acidotic conditions already at baseline and even more so after 30 min of incubation ex vivo. *p < 0.05; **p < 0.01; ***p < 0.001; ****p < 0.0001; ns: not significant; MPC: 2-methacryloyloxethyl phosphorylcholine; FPX: fondaparinux; MCF: mean clot firmness; MFI: medium fluorescence intensity. For better visibility, statistically non-significant differences were only marked, if of relevance for the experimental hypothesis.

The ex vivo human whole blood model (hWBM) for this study was previously published^18,19^, with minor modifications as described hereinafter. After sampling, FPX-anticoagulated blood was transferred to 2-methacryloyloxethyl phosphorylcholine polymer (MPC, Lipidure^®^; NOF Corporation, Japan) coated 2 ml tubes. If applicable for the respective experiment, balanced crystalline solution (Jonosterile^®^, Fresenius Kabi, Germany), either neutral or with adjusted pH and/or platelet activating agents, pro-inflammatory cytokines, DAMPs or anaphylatoxins were added (see Suppl. Methods 1 and 2 for details on respective protocols; Figs. 1A and 3A). Then, tubes were gently inverted and placed on a spinning wheel inside a heating cabinet preset to either 37 °C, 34 °C–30 °C and incubated under constant motion for 30–120 min. After incubation, blood from the respective samples was transferred to respective tubes for further analyses, as described below and in the supplementary methods.

Platelet rich plasma (PRP) was generated immediately after blood drawing by centrifuging the sampling monovettes at 150 g for 15 min with lowest possible settings for acceleration and brake and carefully collecting the upper part of the supernatant. PRP was then transferred to MPX coated tubes and processed as described above.

### Blood gas analysis, blood count, ROTEM and ELISA

We performed blood gas and acid-base-status analysis, blood counts measurements and ROTEM analysis from blood samples (see Suppl. Method 5 for details). Commercially available ELISA kits were used to determine concentrations of sCD154, von-Willebrandt factor (vWF) and IL-8 (see Suppl. Method 3 for details).

### Flow cytometry

Surface expression of CD62P, CD63 and CD154 on platelets was analyzed via flow cytometry, either in whole blood, PRP or after isolation from citrated blood, both in resting state and following activation with ADP or thrombin (see Suppl. Method 4 for details).

### Statistics

All data were analyzed and visualized with Prism 10 (GraphPad Software, USA). If applicable, data sets were tested for outliers using the ROUT. Normal distribution was assumed for all data sets, unless stated otherwise. Data was visualized as single values with mean and 95% confidence interval of the mean, unless stated otherwise.

Differences between group means were tested for statistical significance using either t-test (two experimental groups) or repeated measures (RM) one-way ANOVA/Prism 9 mixed-effects model (more than two experimental groups). Analyses were corrected for inequality of variances or missing sphericity, where applicable. Post-hoc tests with correction for multiple comparisons were used as described in the supplementary methods (see Suppl. Statistics). Results were considered statistically significant at p-values < 0.05. The level of significance was depicted as follows: *p < 0.05; **p < 0.01; ***p < 0.001; ****p < 0.0001; ns: not significant. To improve visibility, statistically non-significant differences were only marked, if of relevance for the experimental hypothesis.

## Results

### Acidosis, dilution, and hypothermia exhibit differential, procoagulatory effects in whole blood

The lethal triad (LT) of trauma comprises of acidosis, hypothermia, and (dilutional) coagulopathy. In a first approach, we established a protocol to model increasing grades of acidosis in human whole blood (hWB) and PRP (Fig. 1A/B). We found that acidotic conditions in WB caused grade-dependent platelet activation and (micro-) clotting (Fig. 1C), that correlated well with the pH values (Suppl. Figure 1A). In contrast, in PRP, i.e. in the absence of immune cells, no relevant activation occurred (Fig. 1D).

CD62P, a marker for α-granule secretion following platelet activation, was barely expressed on platelets from °I/°II acidotic hWB, but variably increased after 30 min of °III acidosis (Fig. 1E, left). Subsequent activation with ADP showed intact reaction capacity of platelets after low- and mid-grade acidosis, whereas platelets exposed to °III acidosis did lose their reaction capacity. Similarly, CD63 expression, a surrogate for dense (δ) granule secretion that cannot be initiated by low-dose ADP stimulation, only inconsistently increased after °III acidosis (Fig. 1E, middle). Interestingly, CD154 surface expression on platelets did slightly increased over the three grades of acidosis, both on resting and subsequently stimulated platelets, although the observed changes missed statistical significance (Fig. 1E, right).

In line with platelet counts and activation parameters, acidosis did not alter the expression of the investigated surface markers on resting platelets from acidotic PRP. In contrast, acidosis increased the reaction capacity to ADP stimulation, even for CD63 (Fig. 1F). Of note, some of the °III acidotic hWB sample could not be analyzed by flow cytometry, due to extensive in-tube clotting, which may complicate interpretation of the indicated results. Platelets isolated directly from citrated whole blood did not show any activation or impairment of the corresponding reaction capacity under acidotic conditions (Suppl. Figure 1 C).

Because acidosis grade-dependently increased sCD154 plasma concentration in the hWBM, we further investigated the role of sCD154 para-/autocrine signaling under acidotic conditions. However, neither in the hWBM nor in-vitro (i.e. with directly isolated platelets) did sCD154 exhibit an activating or priming/potentiating effect (Suppl. Figure 2).

ROTEM analyses from acidotic WB did not reveal any changes at low- and midgrade acidosis, whereas °III acidosis significantly impaired clot formation and quality, by means of both the intrinsic and extrinsic pathway (Fig. 1G).

**Fig. 2:**
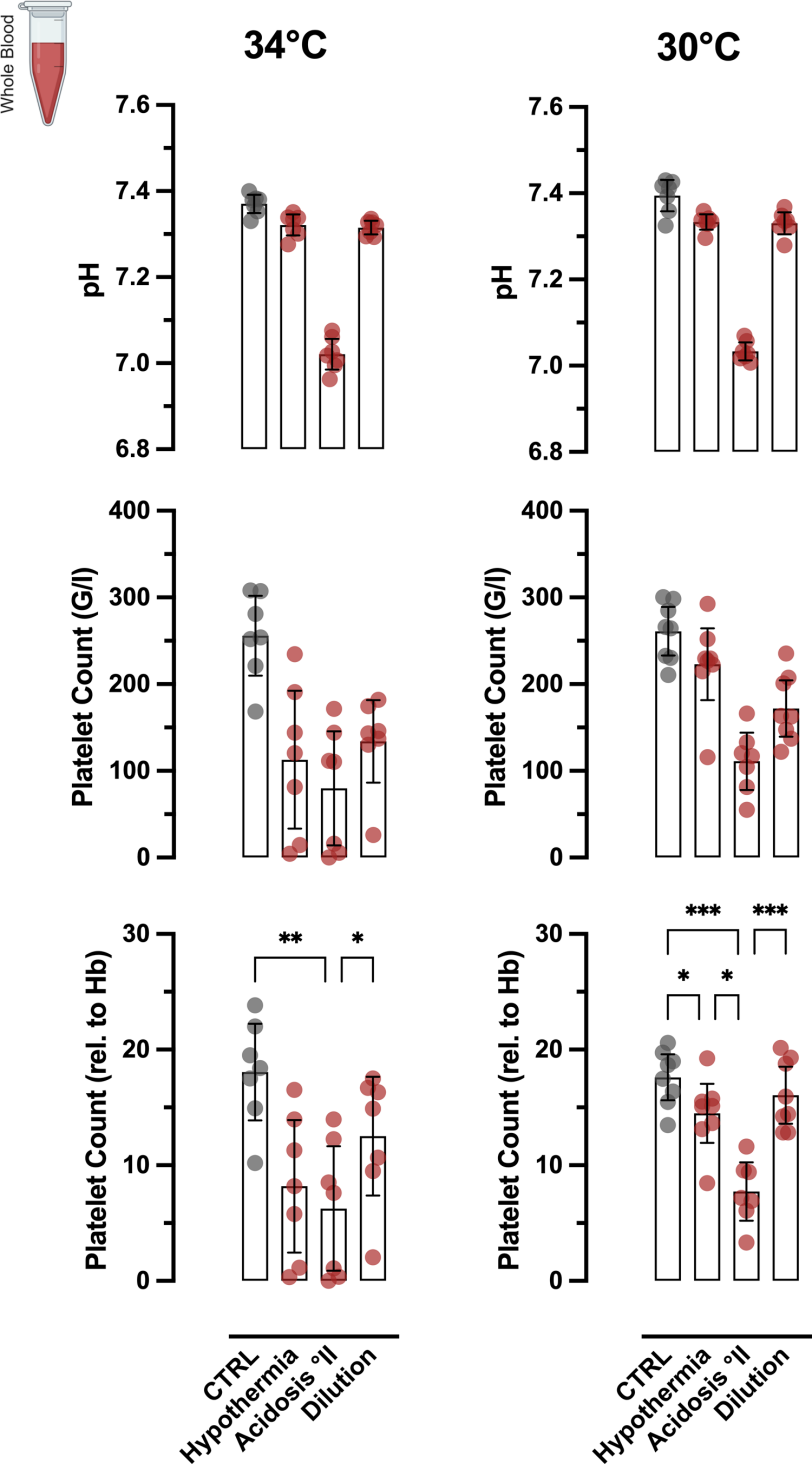
Pro-coagulative effects of hypothermia, acidosis and dilution on platelets in whole blood. Whole blood (hWB) was incubated at 30° and 34° respectively and coagulopathic effects of hypothermia with and without Acidosis °II and 3:7 dilution (jonosteril) were compared with normothermic controls (CTRL). The middle panel displays absolute platelet counts, the lower panel shows counts normalized to hemoglobin concentration to subtract diluting effects. Hypothermia at 34 °C exhibited hypercoagulative effects comparable to °II acidosis. This effect was abrogated at severe hypothermia (30 °C). At mild hypothermia (34 °C), 3:7 dilution with crystalline solution caused a slight drop in platelet counts beyond the mere diluting effect, with in-tube clot formation in 1 sample. This effect was abolished at 30 °C. Acidosis at severe hypothermia caused a more consistent drop in platelets counts but less clotting-formation in tubes after 30 min, suggesting an impaired hemostatic capacity of hWB at lower temperatures. *p < 0.05; **p < 0.01; ***p < 0.001; ****p < 0.0001; ns: not significant. For better visibility, statistically non-significant differences were only marked, if of relevance for the experimental hypothesis.

In a second approach, we established protocols for dilution and hypothermia in WB and PRP. We modelled dilution/consumption coagulopathy by diluting WB and PRP with jonosteril, a balanced crystalline solution. Of note, dilution alone induced a marked, ratio-dependent increase in base deficit but did not affect pH (Suppl. Figure 3 A). We investigated the effects of (central) hypothermia at two grades, mild (34 °C) and severe (30°), both per se and in combination with °II acidosis and dilution (Fig. 2). Notably, hypothermia alone propagated (micro-) clotting and platelet consumption, which was more pronounced, yet more inconsistent at 34 °C, then at 30 °C. Under hypothermic conditions, the effect of °II acidosis was comparable to preceding normothermic experiments. At 34 °C, dilution with jonosteril at a ratio of 3:7 caused a slight drop in relative platelet counts in all samples with even pronounced clotting in one sample. At severe hypothermia, this prothrombotic effect appeared abrogated (Fig. 2).

### The lethal triad further drives hypercoagulative TIC

**Fig. 3:**
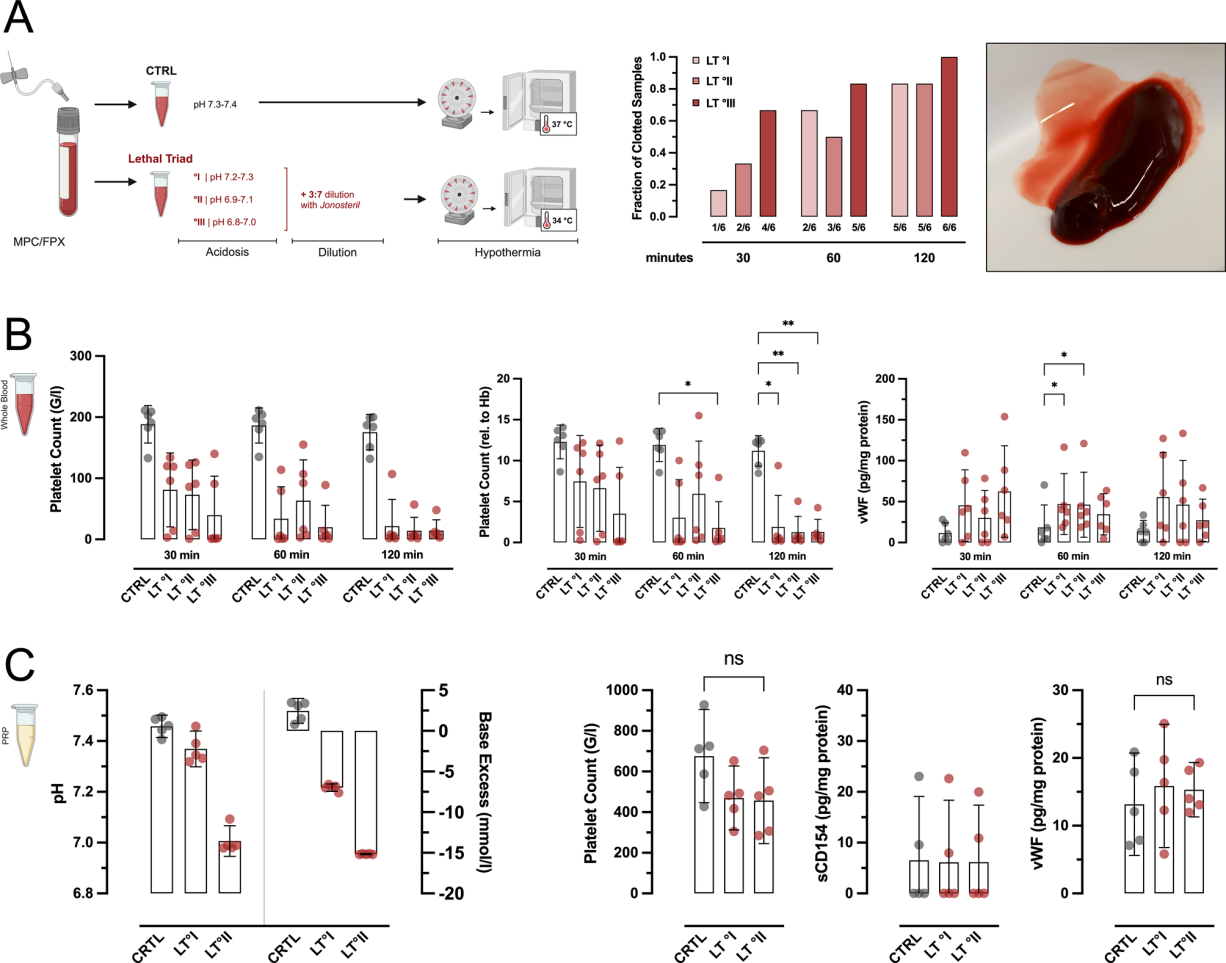
Hypercoagulative effects of the lethal triad in hWB and PRP. (**A**) Protocol for the lethal triad (LT) in human whole blood (hWB) and platelet rich plasma (PRP). LT caused time- and grade-dependent hypercoagulation, with incremental clot formation ex vivo, which was also reflected in decreased relative platelet counts and slightly increased vWF plasma concentration (**B**). As samples with higher grade acidosis and later time-points showed substantial in-tube clotting, only small volumes of plasma could be retrieved. (**C**) in PRP, i.e. in the absence of cellular immunity, LT conditions had no pro-coagulative effect. *p < 0.05; **p < 0.01; ***p < 0.001; ****p < 0.0001; ns: not significant; MPC: 2-methacryloyloxethyl phosphorylcholine; FPX: fondaparinux; Hb: hemoglobin. For better visibility, statistically non-significant differences were only marked, if of relevance for the experimental hypothesis.

After establishing ex-vivo protocols for isolated acidosis, dilution and hypothermia, respectively, our next step was to investigate the triad in regard to TIC-driving effects. We therefore modelled 3 grades of LT (°I-III), defined by increasing severity of acidosis and analyzed platelet activation over 3 time points (Fig. 3A; Suppl Fig. 3B). We observed a time- and grade-dependent hypercoagulatory effect, with incremental ex vivo clot formation (Fig. 3A, right) and thus significant platelet consumption and depletion (Fig. 3B). Of note, vWF concentrations in plasma were only slightly increased (Fig. 3B, right panel) and sCD154 concentrations were unaltered (data not shown). However, due to the substantial in-tube clotting, especially at higher grade and later time points, only small volumes of plasma could be retrieved. Therefore, results need to be interpreted with caution. As shown for acidosis, there was no perceivable activation in PRP (Fig. 3C).

### Histones exponentiate lethal triad-driven hypercoagulative TIC

In a next step, we evaluated potential aggravating effects of cellular damage and systemic inflammation on the LT-driven TIC. We first modelled two grades of systemic inflammation by adding pro-inflammatory cytokines to hWB ex vivo in both presence and absence of a °II LT state. Neither did we observe any additional pro-coagulative effects (Suppl. Figure 4 A), nor any platelet (pre-) activation, assessed by surface expression of CD62P, CD63 or CD154 (Suppl. Figure 4B). Likewise, neither HMGB1, a previously described nuclear DAMP, nor complement anaphylatoxins did further impact ex vivo TIC in hWB or in PRP (Suppl. Figure 4 C/D).

**Fig. 4:**
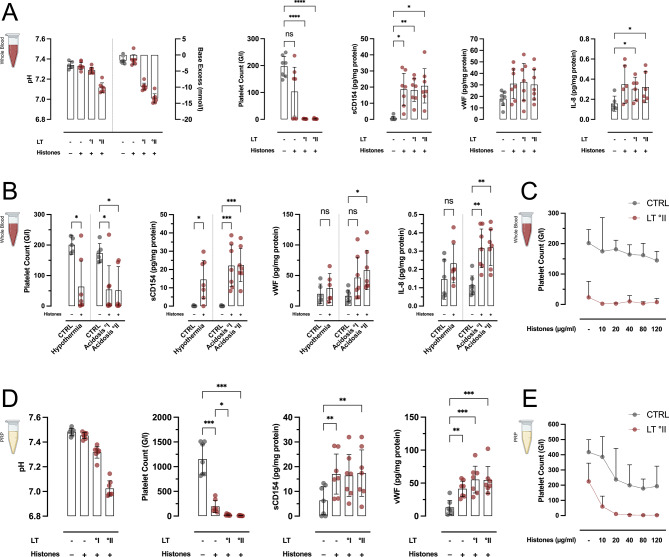
Coagulopathic effect of histones on lethal triad-driven TIC. (**A**) Ex vivo incubation of hWB with 50 µg/ml histones ex vivo caused clot-formation in a fraction of samples. When incubated under LT conditions, histones caused overwhelming platelet activation and clot formation. (**B**) The hypercoagulative effect was slightly less pronounced when incubated under sole hypothermic or acidotic conditions, respectively. (**C**) The effect of histones showed no relevant concentration-dependency in hWB. (**D**) In PRP, the hypercoagulative effect of 50 µg/ml histones was similarly substantial, with (**E**) some concentration-dependency. *p < 0.05; **p < 0.01; ***p < 0.001; ****p < 0.0001; ns: not significant; MPC: 2-methacryloyloxethyl phosphorylcholine; FPX: fondaparinux; Hb: hemoglobin. For better visibility, statistically non-significant differences were only marked, if of relevance for the experimental hypothesis.

Histones–nuclear DNA-associated proteins—are well characterized DAMPs, that have been described to activate cellular hemostasis^20^. In hWB, histones alone propagated platelet activation and micro-clotting, albeit with some donor-specific variability. Remarkably, when acting under LT conditions, histones caused an overwhelming hypercoagulative effect, with complete in-tube clotting of all samples, even after 30 min (Fig. 4A). This effect was somewhat diminished, when histones were combined with isolated hypothermia or acidosis, respectively (Fig. 4B). In hWB, the striking impact of histones on TIC was not concentration-dependent (Fig. 4C). In contrast to our observations regarding the ex vivo incubation of PRP under LT conditions, the histones also induced substantial clot-formation in the absence of cellular immunity (Fig. 4D). This hypercoagulative effect appeared to be at least in part concentration-dependent (Fig. 4E).

## Discussion

Hemorrhagic shock is widely recognized as key driver of posttraumatic multiple organ failure, including coagulopathy^21^. Main pathophysiological triggers are the reduction in cellular oxygen supply to the endothelium, and generation of deleterious reactive oxygen species during the reperfusion phase. Clinically, bleeding with development of acute coagulopathy remains among the most preventable conditions following severe tissue trauma^22^. Nevertheless, the underlying mechanisms and potential treatment targets are still a matter of debate. Utilizing an ex vivo human whole blood lethal triad model, we observed that even small shifts in the pH led to reductions in thrombocyte count. Severe acidotic conditions halved the thrombocyte number and elicited the major platelet activation markers.

Platelets are especially a rich source of CD154 (within the α-granula), which as a member of the TNF family represents the ligand for CD40. The acidotic (micro-) environment seems to activate platelets, subsequently releasing the soluble form sCD154. This may orchestrate humoral immunity to turn an inert endothelial surface to a proadhesive, proinflammatory, and procoagulatory endothelial phenotype, characterized for example by enhanced tissue factor (TF) expression^23^. Moreover, sCD154 autoactivates platelets via feed-back amplification, further promoting a procoagulatory state. Whether sCD154 release stems directly from pH alterations or from cleavage processes by intermediary proteases, such as metalloproteases, warrants further exploration. Similarly, the platelet-derived vWF (in absence of the endothelium) was significantly increased in response to pH reduction, contributing to a procoagulatory milieu^23^. Endothelial vWF, under acidic conditions (at pH values around 6.5), undergoes structural changes, aggregating at the cell surface and failing to form filamentous strands in response to fluid shear, thereby exacerbating coagulopathic features^24^. However, as a major limitation of the study, the current model neither includes the endothelium nor components of the luminal glycocalyx layer. Shock-induced activation of metalloproteinases with subsequent glycocalyx shedding leads not only to autotransfusion but also to auto-dilution, and auto-heparinization effects, shifting the endothelium from anti-thrombotic, anti-adhesive, anti-inflammatory to pro-coagulatory, pro-adhesive, pro-inflammatory state^25,26^. Thus, future analyses should incorporate the endothelium to enhance translational relevance.

The acidotic shift influenced the hemostatic cascade, as evidenced by enhanced clot formation time and time to mean clot firmness, as well as decreased alpha-angle in the thromboelastographic analyses. These findings are supported by previous reports^27^ indicating direct effects of the pH on fluid and cellular phase of coagulation. For example, a pH shift to 7.0 decreased the rate constants for thrombin and FXa inhibition by ca. 30%, although overall dynamics of clot formation were slightly increased^28^. However, correcting pH alone failed to reverse acidosis-induced coagulopathic states, highlighting the complex molecular interplay involved^29^. Of note, for methodical reasons we did not induce acidosis via adding lactic acid, and therefore, our model does not fully reflect any additional pathophysiological effects by lactate build-up from hypoxic tissue. However, as previously reported^18^, there is an increase in lactate in our ex vivo model over time, which may have contributed to the time-dependent effects seen under lethal triad conditions.

Another confounding factor after severe tissue injury, is accidental hypothermia (i.e. core temperature below 35 °C) which is simple to measure and monitor but complex to understand. Such hypothermic conditions exacerbate coagulatory and inflammatory complications and significantly increase mortality rates^30^. Mild hypothermia between 32 and 35 °C, commonly observed in polytrauma patients^31^, is associated with vasoconstriction, increased oxygen consumption, tachypnoea, platelet dysfunction, and impaired clotting factor activity^31^. On the molecular level, hypothermia of 32 °C comprises the initial phase of thrombin production and fibrinogen synthesis, whereas acidosis inhibits thrombin generation and promotes fibrinogen degradation, indicating differential mechanisms, which however all add to the development of coagulopathic states^16^. In a rodent model of mild hypothermia (33–34 °C) there was no difference in the coagulatory capacity in comparison to normothermic littermates; nevertheless, in case of an additional traumatic hemorrhagic shock, hypothermia prolonged clotting and weakened clot firmness^32^. In contrast, application of a therapeutic mild hypothermia (34 °C) after porcine polytrauma revealed some minor effects on the coagulation but no aggravation of the trauma-induced coagulopathy^33^. Therefore, Mohr et al. concluded that mild hypothermia was safe in the context of polytrauma^33^. While the impairing effect of hypothermia on hemostasis is well-established in the literature, our data indicate a more nuanced impact of progressive hypothermia on post-traumatic coagulopathy.

To simulate the complex clinical reality of the lethal triad, our model incorporated iatrogenic dilution, revealing significant platelet count drops and increased platelet activation markers. A more severe lethal triad model (33% dilution, pH 6.8, 32 °C) exposed to citrated blood demonstrated similar platelet count drops and activation, reflected by CD42b^+^/CD62P^+^ expression^34^, emphasizing the validity of our model. Notably, in the present human whole blood model, the overall function of the clotting system remained fully intact as -in contrast to other ex vivo blood models- only a minor intervention in the clotting system was necessary (specific factor Xa inhibition with FPX at relatively low concentrations). This dynamic functionality could be achieved by coating the tubes with 2-methacryloyloxethyl phosphorylcholine (MPC) reliably preventing surface deposition of blood proteins and subsequent clot formation as previously demonstrated^18^. Thereby, in our present model, the fluid and cellular phase of the thrombo-inflammatory systems including the coagulation system and the complement system, could be functionally preserved accompanied by a stable physiological and metabolic environment. This allowed to investigate especially the additional effects of DAMP exposure.

Histones acting as DAMPs elicited clot formation and platelet consumption at concentrations, comparable to those of trauma patients^35^. Moreover, in the presence of the modelled lethal triad, histone exposure proved to be more relevant in reducing platelet count than a pathophysiological environment in the absence of histones. In mice, histones bound to platelets, triggered a calcium influx and fibrinogen recruitment, which in turn culminated in platelet aggregation and a subsequent drop in platelet count^20^. However, the inhibitory effect of albumin on the histone-caused platelet decline^36^ necessitated markedly higher concentrations of histones^20^ than those observed in present model. This suggests a role of albumin as “scavenger” for circulating histones, that could be compromised by dilution and/or increased binding of albumin to free hydrogen protons in an acidotic environment as present in our novel model. Histones also resulted in enhanced vWF concentrations in the blood even in absence of endothelial structures. Additionally, histones spurred an increase in sCD154 levels in both whole blood and PRP, indicating platelets as a primary source rather than lymphocytes. CD154, in turn, may trigger TF generation in endothelial and smooth muscle cells thereby shifting them towards a thromboinflammatory state^37^. In contrast, HMGB-1 did not significantly influence coagulation properties. Neither did complement anaphylatoxins C3a and C5a, thus supporting recent findings on the crosstalk of complement and hemostasis^19^. Collectively, histones as DAMPs may represent a crucial contributing factor to the lethal triad. These findings may pave the way for novel therapeutic approaches to lethal triad driven TIC. Such approaches may include careful correction of the acidosis and even albumin substitution to account for potential scavenging effects as mentioned above. Furthermore, previous studies have shown beneficial effects of neutralizing antibodies targeting histones in experimental trauma^35^ and a plethora of therapeutic strategies have been proposed to ameliorate histone effects in states of inflammation (see^38^ for a review on the topic). However, future studies should not only focus on histones but also address hypocalcemia, which has been proposed as lethal diamond^39^, given its association with coagulopathy and poor outcome. Whether it is time to denote the presence of histones as the “lethal pentagon” warrants further exploration, particularly within the clinical context.

## Electronic supplementary material

Below is the link to the electronic supplementary material.

Supplementary Material 1

## Data Availability

The datasets used and/or analyzed during the current study available from the corresponding author on reasonable request.
