# Supplementary material for: Histone-driven hypercoagulation contributes to the lethal triad of acute trauma-induced coagulopathy
Source: Sci Rep. 2025 Aug 19;15:30422. doi: 10.1038/s41598-025-12431-7 (PMC12365074; doi:10.1038/s41598-025-12431-7)
Supplement: Supplementary file 1 — Supplementary Material 1 [file 41598_2025_12431_MOESM1_ESM.pdf]

Lethal Triad revisited: Differential impact of pathophysiological conditions and danger molecules on Acute Trauma-Induced Coagulopathy (TIC)

Christian Karl Braun[1], Marco Mannes[1], Doreen Spiegelburg[1], Frederik Müller[1], Amadeo Klitzing[1], Anke Schultze[1], Gerhard Achatz[2], Andreas Bauer[2], Markus Huber-Lang[1]

[1] Institute for Clinical and Experimental Trauma-Immunology, Ulm University Medical Center, Ulm, Germany

[2] Department of Trauma Surgery and Orthopaedics, Reconstructive and Septic Surgery, Sportstraumatology, Trauma Surgery Research Group, German Armed Forces Hospital, Ulm, Germany

Supplemental Data

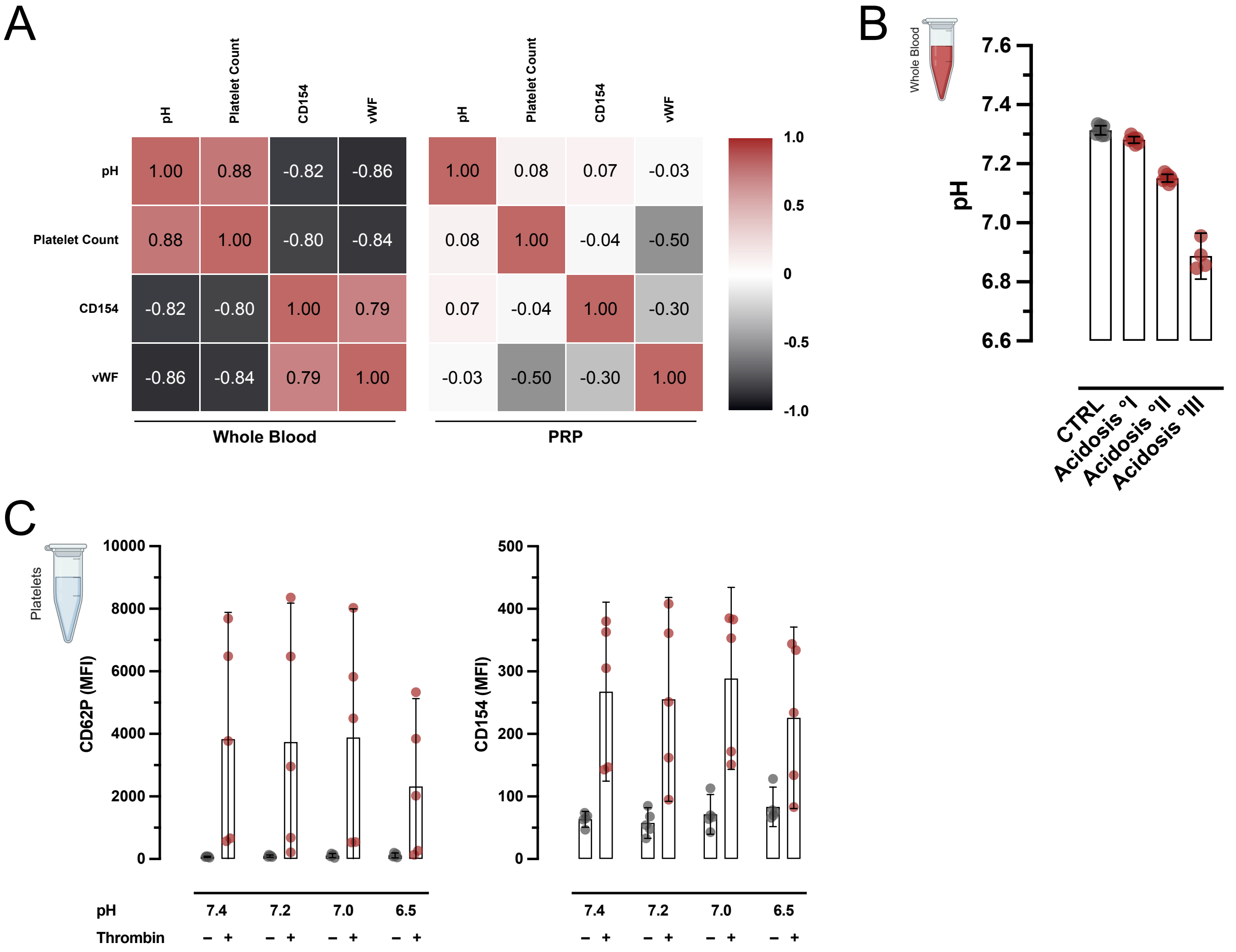

Suppl. Fig. 1
A) Correlation matrix of pH and surrogates of platelet activation after 30 min of modelling acidosis, both in hWB and in PRP (Pearson's r is shown for each correlation). B) pH in whole blood from flow cytometer/ROTEM experiments. C) In vitro stimulation of platelets with low doses of thrombin (0.2 U/ml) under acidic conditions showed sustained reaction capacity. A sub-maximal dose of thrombin was used, to account for even small changes due to donor specific idiosyncratic reaction capacity of platelets.

For better visibility, statistically non-significant differences were only marked, if of relevance for the experimental hypothesis.

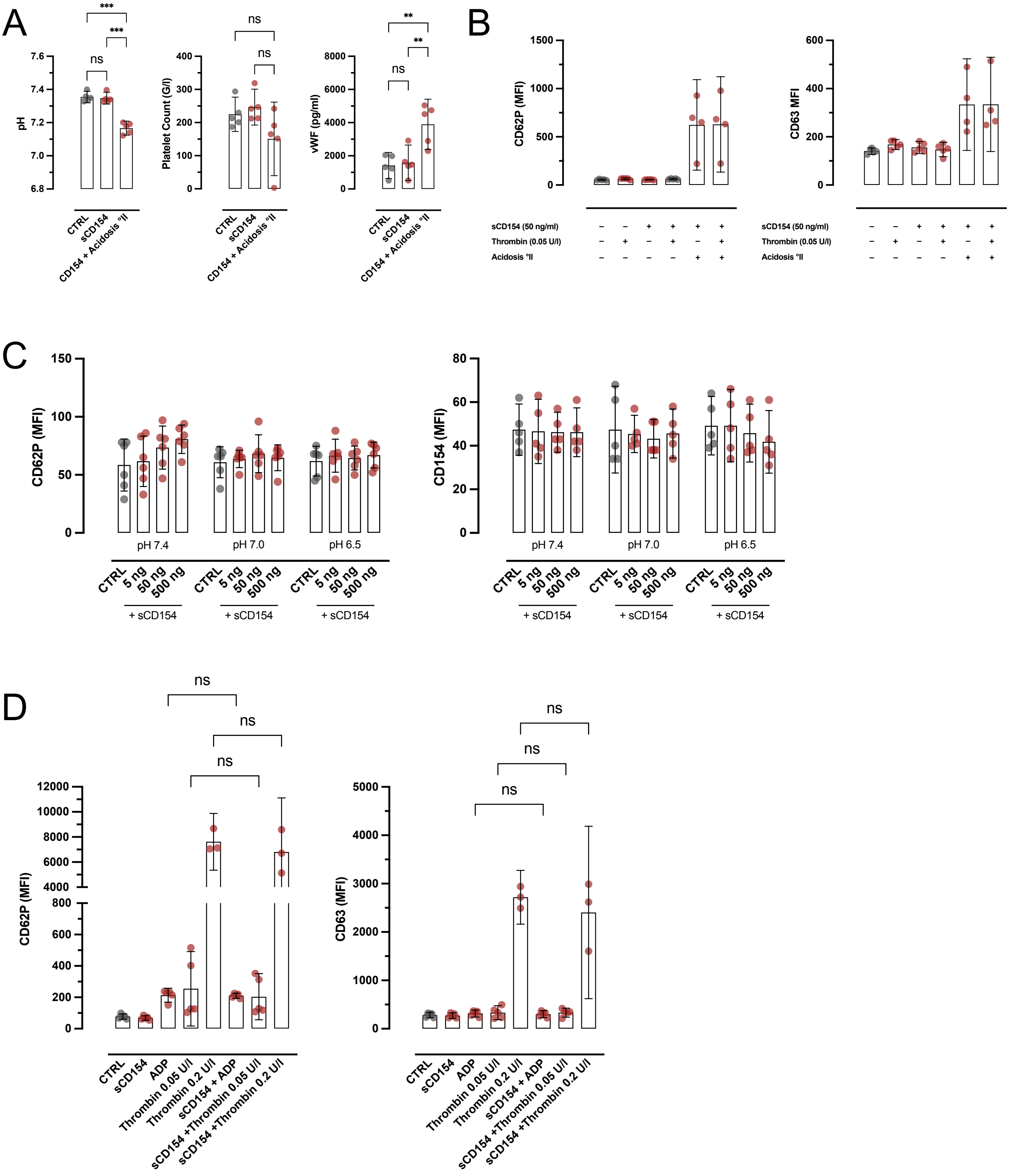

Suppl. Fig. 2

A) Impact of stimulation with 50 ng/ml of sCD154 in the presence or absence of acidotic condition in hWB. No additional pro-coagulative effects were oberseved. B) Flowcytometric analysis of platelets from hWB showed no relevant activation or “priming”-effect after 30 min if incubation with sCD154. C) sCD154 stimulation of isolated platelets showed no dose-dependent or acidosis-altered effects. B) Flowcytometric analysis of directly isolated platelets revealed no relevant activation or “priming”-effect of sCD154.

*p<0.05; **p<0.01; ***p<0.001; ****p<0.0001; ns: not significant;
For better visibility, statistically non-significant differences were only marked, if of relevance for the experimental hypothesis.

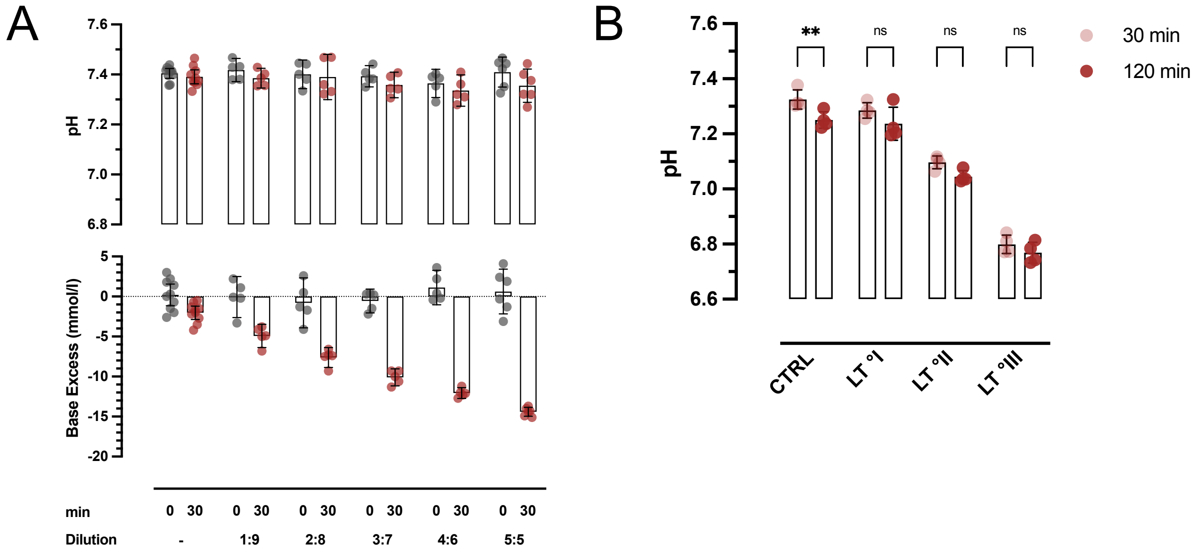

Suppl. Fig. 3
A) Impact of increasing dilution with jonosteril on acid-base status in hWB. Dilution ratio-dependently increased base deficit while pH remained unaltered. B) pH in hWB under LT conditions after 30 min and 120 min of ex vivo incubation respectively.

*p<0.05; **p<0.01; ***p<0.001; ****p<0.0001; ns: not significant;

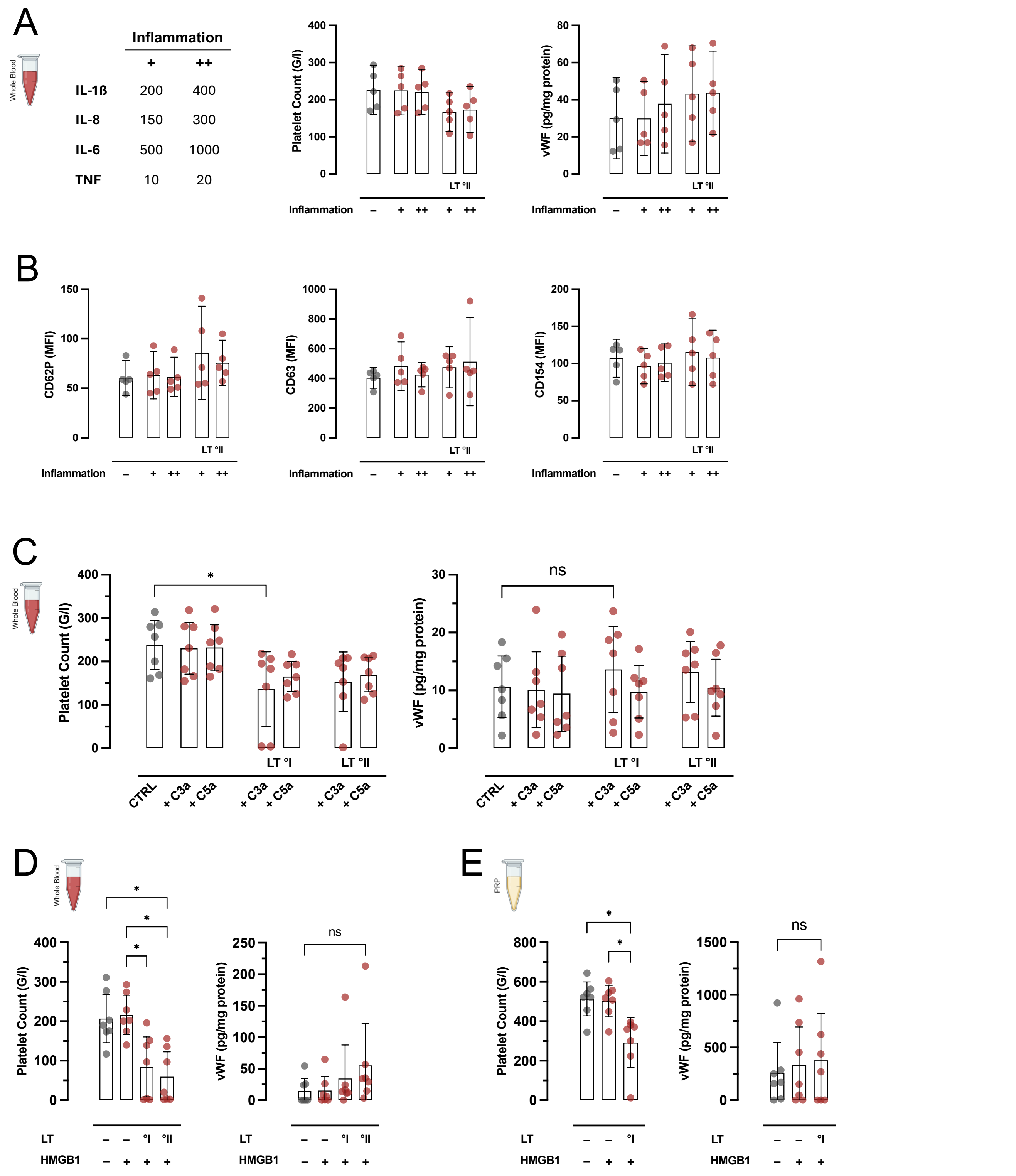

Suppl. Fig. 4
A) Modelling systemic inflammation under LT conditions at two severity grades did not show any TIC-driving effect, neither regarding platelet counts and vWF, nor (B) flowcytometric analysis of surface expression of CD62p, CD63 or CD154. C) Anaphylatoxins (C3a, C5a) did not reveal substantial TIC-driving effects in hWB under LT conditions. D) HMGB1 had no aggravating effect on LT-driven TIC, neither in hWB nor in (E) PRP.

*p<0.05; **p<0.01; ***p<0.001; ****p<0.0001; ns: not significant;

Lethal Triad revisited: Differential impact of pathophysiological conditions and danger molecules on Acute Trauma-Induced Coagulopathy (TIC)

Christian Karl Braun[1], Marco Mannes[1], Doreen Spiegelburg[1], Frederik Müller[1], Amadeo Klitzing[1], Anke Schultze[1], Gerhard Achatz[2], Andreas Bauer[2], Markus Huber-Lang[1]

[1] Institute for Clinical and Experimental Trauma-Immunology, Ulm University Medical Center, Ulm, Germany

[2] Department of Trauma Surgery and Orthopaedics, Reconstructive and Septic Surgery, Sportstraumatology, Trauma Surgery Research Group, German Armed Forces Hospital, Ulm, Germany

Supplemental Methods

Suppl. Methods 1 – Models of Lethal Triad in human Whole Blood and PRP

Acidosis in Whole Blood and PRP

To model metabolic acidosis in whole blood and PRP, pH-adjusted crystalline solution (Jonosteril®, Fresenius Kabi, Germany) was added to the freshly drawn blood, and samples were incubated for 30 min under constant motion at 37°C (Fig. 1 A). Aliquots of Jonosteril were titrated with HCl 3.7% to a pH of 0.5, 1.0 and 3.0, respectively. Either unadjusted Jonosteril (pH ~7.0; controls) or pre-adjusted Jonosteril (pH 3.0 for °I; pH 1.0 for °II; pH 0.5 for °III) was preloaded to MPC coated 2 ml tubes and freshly drawn, FPX anticoagulated whole blood was added to final volume ratio of 25 µl/ml. Tubes were then carefully inverted, immediately placed on a spinning wheel inside a heating cabinet at 37°C and incubated for 30 min. After incubation, samples were processed for respective analyses as described in *methods*.

Dilution and Hypothermia in Whole Blood and PRP

Consumption coagulopathy (through micro-clotting) and (iatrogenic) blood dilution plays an important part in TIC development. We modelled intravascular dilution, as it is occurring through volume shift from the extra-vascular/extra-cellular space ("autoinfusion") and through therapeutic fluid-resuscitation above-mentioned hemorrhagic shock, via addition of jonosteril (see above). For modelling the lethal triad, we added 250 µl of jonosteril (unadjusted, pH ~7.0) plus 50 µl of pH-adjusted jonosteril (see above) per 700 µl of FPX anticoagulated whole blood or PRP, to yield a volume ratio of 3:7. In adults, this ratio reflects a clinically relevant blood loss or rather fluid resuscitation regime of roughly 1.5-2 l of crystalline solution in the first 30 min.

Central hypothermia, as a crucial part of the lethal triad, was modelled via hWB/PRP incubation in a heating cabinet at 30°C or 34 °C, respectively (Fig. 2A).

Lethal Triad in Whole Blood and PRP

The above-mentioned protocols were merged, to model the lethal triad (LT), both in hWB and PRP. Throughout, dilution was modelled at a ratio 3:7 and central hypothermia was modelled at 34°C. LT grades (°I-III) were determined through increasing grades of acidosis (Fig. 2A), accounting for the substantial impact of acidosis observed in preceding experiments. For that, 50 µl of pH-adjusted jonosteril(°I: pH 3; °II: pH 1; °I: pH 0.5) were added to 250 µl of unadjusted (pH ~7.0) jonosteril and pre-loaded to 2 ml tubes. FPX anticoagulated hWB or PRP were added, tubes were gently inverted and incubated at 34°C for up to 120 min under constant motion. After incubation, blood samples were immediately processed as described in respective sections.

Suppl. Methods 2 – Activating Agents in hWB and PRP

sCD154 Stimulation in hWB

Soluble CD154 (sCD154 = sCD40L; Cat-No.: ab179625; Abcam, United Kingdom) was added to 50 µl of either neutral or pH-adjusted jonosteril (pH 1.0; °II acidosis) to yield a final (i.e. in hWB) concentration of 50 ng/ml. hWB samples were then incubated at 37°C for 30 min, as described above.

Modeling systemic Inflammation in hWB

To investigate additional TIC-driving effects of sterile inflammation, as observed early after severe trauma and hemorrhagic shock, we modelled to two grades of systemic inflammation (cf. Fig. 3A). IL-1β (Cat.-No.: 17810723; Gibco™ Human IL-1 beta Recombinant Protein, PeproTech®; Thermo Fisher, USA; final concentration: 200/400 pg/ml), IL-6 (Cat.-No.: 348148.5; Interleukin-6, recombinant, human; Biomol, Germany; final concentration: 500/1000 pg/ml), IL-8 (Cat.-No.: 348154.5; Interleukin-8/CXCL8, recombinant, human; Biomol, Germany; final concentration: 150/300 pg/ml) and TNFα (Cat.-No.: GF314; TNF- α Protein, Human Recombinant Animal Free; Sigma Aldrich/Merck, Germany; final concentration: 10/20 pg/ml) were mixed at two different concentrations (inflammation +/++; Fig. 3A) in $PBS^{-/-}$. 200 µl stock mixture were added to 1.8 ml of hWB and samples were incubated for 30 min at 37°C under constant motion. To model inflammation under lethal triad °II conditions, 200 µl of stock mixture were added to 400 µl of pH-adjusted jonosteril, pre-loaded for 1.4 ml of hWB and samples were incubated at 34°C as described above.

DAMP and Anaphylatoxin Stimulation in hWB and PRP

We analyzed coagulopathic effects of DAMPS and activated complement components in the context of lethal triad conditions. Either Histones (from calf thymus; Cat.-No.: 10 223 565 001; Roche Diagnostics, Germany; final concentration: 50 µg/ml, unless stated otherwise), HMGB1 (recombinant; Cat.-No.: 1690-HMB; R&D Systems, USA; final concentration: 500 ng/ml) or anaphylatoxins C3a and C5a (both CompTech, USA; final concentration C3a: 500 ng/ml; final concentration C5a: 10 ng/ml) were added to jonosteril for a final volume of 50 µl per 1 ml hWB, respectively, and preloaded to 2 ml tubes. For combination with lethal triad conditions, agents were added to 50 µl of pH-adjusted jonosteril and added to 250 µl of neutral jonosteril and preloaded to 2 ml tubes. Respective volumes of freshly drawn, FPX-anticoagulated hWB or RPR for a final in-tube volume of 2 ml were added and the samples were incubated under constant motion, as described above.

Suppl. Methods 3 – ELISA Kits and total Protein Concentration

ELISA Sample Preparation

After incubation, whole blood and PRP was mixed with EDTA to a final concentration of 10 mM and centrifuged at 800 g for 5 min at 4°C. Supernatant fluid was collected and centrifuged again at 16.000 g for 2 min at 4°C. Plasma samples were stored at -80°C until used. Commercially available ELISA kits were used to determine the plasma concentrations of sCD154, von-Willebrandt factor (vWF) and IL-8 (Suppl. Method 3).

ELISA Kits

The following, commercially available ELISA kits were used, strictly adhering to the manufacturer's instructions: sCD154 (i.e. sCD40L, Human CD40 Ligand DuoSet; DY617, R&D systems, USA), von-Willebrandt factor (Human vWF-A2 DuoSet; DY27664-05, R&D systems, USA) and IL-8 (Human IL-8 DuoSet ELISA; DY208, R&D systems, USA), adhering to the manufacturer's instructions. Absorption was measured with a Tecan Sunrise Reader (Tecan Tradding AG, Switzerland) at 450 nm wavelength and total concentrations were calculated from a standard curve.

Determination of total Protein Concentration in Plasma Samples

To account for dilution effects in the lethal triad models, plasma concentrations were related to total protein levels in each sample. Total protein concentrations were measured, using the Pierce BCA Protein Assay Kit (Cat.-No.: 23225; Thermo Fisher, USA), adhering to manufacturer's instructions. In short, plasma samples were diluted 1:100 in de-ionized water and mixed with the reaction reagents and incubated for 30 min at 37°C. Standards were prepared according to manufacturer's instructions. Absorption was measured with the Tecan Sunrise Reader at 562 nm wavelength.

Suppl. Methods 4 – Flow Cytometry

Flow Cytometry of Platelets from Whole Blood and PRP

Aliquots from whole blood and PRP samples were diluted 1:12.5 in pre-warmed HBSS$^{-/-}$ buffer solution after respective incubation. Then, either PBS$^{-/-}$ (controls), ADP (Cat.-No.: A2754; Sigma Aldrich, Germany; final concentration: 5 µM) or thrombin (Cat.-No.: 605190; Merck, Germany; final concentration: 0.05 - 0.2 - 1 U/ml, respectively) were added to 40 µl of each respective sample, mixed and incubated for 10 min at 37°C.

After that, samples were stained with anti-CD41 (250 ng/ml; Cat.-No.: 303710; BioLegend, USA; isotype: 400122, BioLegend, USA), anti-CD62P (2 µg/ml; Cat.-No.: 304904; BioLegend, USA; isotype: 400108, BioLegend, USA), anti-CD63 (1 µg/ml; Cat.-No.: 353004; BioLegend, USA; isotype: 400113, Biolegend, USA) and anti-CD154 (0,5 µg/ml; Cat.-No.: 310846; BioLegend, USA; isotype: 400144 , BioLegend, USA) for another 5 min at 37°C. Subsequently, samples were transferred to tubes containing 2 ml of HBSS$^{-/-}$ buffer solution and finally diluted 1:2 with HBSS$^{-/-}$ buffer solution in FACS tubes. Cells were analyzed with the FACSLyric 2 (BD, USA).

Flow Cytometry of isolated Platelets

Blood was directly drawn into neutral monovettes pre-loaded with citrate-phosphate-dextrose buffer (CPD; 16 mM citric acid, 90 mM NaCl, 16 mM Na2HPO4, 142 mM glucose, pH 7.4) and centrifuged at 150 g for 15 min with lowest possible settings for acceleration and brake. 45 µl of the supernatant PRP were collected, mixed 1:1 with HEP buffer (140 mM NaCl, 2.7 mM KCl, 3.8 mM HEPES, 5 mM EGTA, pH 7.4) and then iloprost (SML1651, Sigma Aldrich, Germany) was added to a final concentration of 10 µM.

Samples were centrifuged at 100 g for 15 min, supernatant was discarded, and pellets were rinsed with platelet-wash-buffer (10 mM sodium citrate, 150 mM NaCl, 1 mM EDTA, 1 % (w/v) glucose, pH 7.4) and eventually resuspended in tyrode's buffer (134 mM NaCl, 12 mM NaHCO3, 2.9 mM KCl, 0.34 mM Na2HPO4, 1 mM MgCl2, 10 mM HEPES, pH 7.4). For acidosis experiments with isolated platelets, Tyrode's buffer was adjusted to decreasing pH (7.4 - 7.2 - 7.0 - 6.5) with HCl.

40 µl of the resulting suspension were mixed with either PBS$^{-/-}$ (controls), ADP, thrombin, sCD154 (Cat-No.: ab179625; Abcam, United Kingdom; final concentration: 5 - 50 - 500 ng/l , respectively) or Jonosteril (with pH adjusted to 0.5/1/3, respectively) and incubated for 10 min at 37°C. After incubation, samples were stained as described above, fixed with 0.5 % PFA for another 10 min, centrifuged at 300 g for 10 min and resuspended in 500 µl PBS$^{-/-}$. Samples were transferred to FACS tubes, diluted 1:1 in PBS$^{-/-}$ analyzed.

Gating Strategy

Platelets were gated as described before [1]. In brief, singlets were identified in a FSC-H/FSC-A plot and a platelet population was identified by size in the FSC-A/SSC-A plot. CD41+ platelets were examined for CD62P, CD63 or CD154 expression respectively.

Suppl. Methods 5 – Blood Gas Analyses, Blood Count and ROTEM Analyses.

Blood Gas Analysis and Blood Count

For blood gas and acid-base-status analysis, blood was collected in heparinized capillary tubes after incubation and measured with the ABL800 flex analyzer (Radiometer Medical ApS, Germany). Small blood count was determined by the certified routine laboratory of the Ulm University Medical Center from human whole blood (hWB) or PRP aliquots collected in EDTA monovettes after incubation.

ROTEM

Whole blood samples were analyzed with rotational thromboelastometry (ROTEM; ROTEM® delta, Tem Innovations, Germany) to assess extrinsic and intrinsic coagulation. After incubation, blood was citrated, kept at room temperature and measured within 1 h. Samples were first recalcified with star-tem 20 and then activated with either ex-tem or in-tem reagents, respectively (all Tem Innovations, Germany).

Literature

[1] Mannes M, Pechtl V, Hafner S, Dopler A, Eriksson O, Manivel VA, Wohlgemuth L, Messerer DAC, Schrezenmeier H, Ekdahl KN et al: Complement and platelets: prothrombotic cell activation requires membrane attack complex-induced release of danger signals. Blood Adv 2023, 7(20):6367-6380.

Lethal Triad revisited: Differential impact of pathophysiological conditions and danger molecules on Acute Trauma-Induced Coagulopathy (TIC)

Christian Karl Braun[1], Marco Mannes[1], Doreen Spiegelburg[1], Frederik Müller[1], Amadeo Klitzing[1], Anke Schultze[1], Gerhard Achatz[2], Andreas Bauer[2], Markus Huber-Lang[1]

[1] Institute for Clinical and Experimental Trauma-Immunology, Ulm University Medical Center, Ulm, Germany

[2] Department of Trauma Surgery and Orthopaedics, Reconstructive and Septic Surgery, Sportstraumatology, Trauma Surgery Research Group, German Armed Forces Hospital, Ulm, Germany

Supplemental Statistics

Fig. 1 - Statistical Analyses

Figure 1C
No outliers; n=7 for each group.
Statistical analysis: repeated measures one-way ANOVA with Geisser-Greenhouse-correction.
Post-hoc test: Tukey's multiple comparisons test; all group pairs tested.

Figure 1D
No outliers; n=7 for each group, except sCD154 data set: 1 donor removed from all groups, due to technical reasons (n=6 for each group).
Statistical analysis: repeated measures one-way ANOVA with Geisser-Greenhouse-correction.
Post-hoc test: Tukey's multiple comparisons test; all group pairs tested

Figure 1E
1 outlier removed (ROUT method; Q1%) from: CD62p | Acidosis °II/–ADP: MFI 406.
n=8 except for:
CTRL/–ADP and CTRL/+ADP: n=7 each, due to handling error of sample;
°III/–ADP and °III/+ADP: n=5 each, due to in-tube clotting during incubation;
Statistical analysis: Prism 10 Mixed Effects Model with Geisser-Greenhouse-correction.
Post-hoc test: Šídák's multiple comparisons test; following the experimental hypothesis, only group pairs of interest were tested for significance;

Figure 1F
No outliers;
n=5 except for:
°I/–ADP and °I/+ADP: n=4 each, due to extensive in-tube clotting during incubation;
°II/–ADP and °II/+ADP: n=4 each, due to extensive in-tube clotting during incubation;
Statistical analysis: Prism 10 Mixed Effects Model with Geisser-Greenhouse-correction.
Post-hoc test: Šídák's multiple comparisons test; following the experimental hypothesis, only group pairs of interest were tested for significance;

Figure 1G
No outliers;
n=5 except for:
CTRL and °III at baseline: n=4 each, due to handling error of sample;
CTRL and °III at 30 min: n=4 each, due to handling error of sample;
Statistical analysis: CTRL/Acidosis °I/Acidosis °II, CTRL/Acidosis °III at Baseline and CTRL/Acidosis °III at 30 min reflect separate experiments (separated in the graph by dotted lines). These experiments were carried out separately to minimize idle time of samples at room temperature. CTRL/Acidosis °I/Acidosis °II tested using the repeated measures one-way ANOVA or Prism 10 Mixed Effects Model with Geisser-Greenhouse-correction and Tukey's post-hoc multiple comparisons test. CTRL/Acidosis °III at Baseline and CTRL/Acidosis °III at 30 min tested with paired t-test.

Suppl. Fig. 1 - Statistical Analyses

Suppl. Fig 1A
Pearson correlation of data from Fig. 1C (Whole Blood) and 1D (PRP). Pearson's r is shown and color-coded as depicted.

Suppl. Fig 1B
Data for reference only; no experimental hypothesis, therefore no statistical testing performed;

Suppl. Fig 1C
No outliers; n=5 for each group.
Statistical analysis: repeated measures one-way ANOVA with Geisser-Greenhouse-correction.
Post-hoc test: Šídák's multiple comparisons test; following the experimental hypothesis, only group pairs of interest were tested for significance;

Suppl. Fig. 2 - Statistical Analyses
Suppl. Fig 2A
No outliers; n=5 for each group.
Statistical analysis: repeated measures one-way ANOVA with Geisser-Greenhouse-correction.
Post-hoc test: Tukey's multiple comparisons test; all group pairs tested.

Suppl. Fig 2B
No outliers;
n=5 except for:
Acidosis °II/+sCD154/–Thrombin and Acidosis °II/+sCD154/+Thrombin: n=4, due to in-tube clotting during incubation;
Statistical analysis: Prism 10 Mixed Effects Model with Geisser-Greenhouse-correction.
Post-hoc test: Tukey's multiple comparisons test; all group pairs tested.

Suppl. Fig 2C
No outliers; n=6 for CD62p, n=5 for CD154;
Statistical analysis: repeated measures one-way ANOVA with Geisser-Greenhouse-correction.
Post-hoc test: Tukey's multiple comparisons test; all group pairs tested

Suppl. Fig 2D
No outliers;
n=5 except for:
Thrombin (0.2 U/ml) and Thrombin (0.2 U/ml) + sCD154: n=3
Statistical analysis: Prism 10 Mixed Effects Model with Geisser-Greenhouse-correction.
Post-hoc test: Šídák's multiple comparisons test; following the experimental hypothesis, only group pairs of interest were tested for significance;

Fig. 2 - Statistical Analyses
Figure 2
No outliers;
n=8 except for:
Acidosis °II at 30°: n=7, due to extensive in-tube clotting during incubation;
Statistical analysis:
pH: data for reference only; no experimental hypothesis, therefore no statistical testing performed;
Platelet counts (absolute, G/l): data for reference only; no experimental hypothesis, therefore no statistical testing performed;
Platelet counts (rel. to Hb): repeated measures one-way ANOVA (34°C) and Prism 10 Mixed Effects Model (30°) with Geisser-Greenhouse-correction. Post-hoc test: Tukey's multiple comparisons test; all group pairs tested.

Suppl. Fig. 3 - Statistical Analyses
Suppl. Fig 3A
Data for reference only; no experimental hypothesis, therefore no statistical testing performed;

Suppl. Fig 3B
No outliers; n=4 for each group.
Statistical analysis: two-way repeated measures ANOVA.
Post-hoc test: Šídák's multiple comparisons test; only row effects tested;

Fig. 3 - Statistical Analyses
Fig. 3B
No outliers; n=6 for each group.
Platelet Count (absolute, G/l) data: data for reference only; no experimental hypothesis, therefore no statistical testing performed;
Other data sets: separated repeated measures one-way ANOVA with Geisser-Greenhouse-correction fopr each time-point. Post-hoc test: Tukey's multiple comparisons test; all group pairs tested.

Fig. 3C
No outliers; n=5 for each group.
pH/BE data: data for reference only; no experimental hypothesis, therefore no statistical testing performed;
Other data sets: Statistical analysis: one-way ANOVA with Geisser-Greenhouse-correction. Post-hoc test: Tukey's multiple comparisons test; all group pairs tested.

Suppl. Fig. 4 - Statistical Analyses
Suppl. Fig. 4A
No outliers; n=5 for each group.
Statistical analysis: repeated measures one-way ANOVA with Geisser-Greenhouse-correction.
Post-hoc test: Dunnett's multiple comparisons test; all group pairs tested.

Suppl. Fig. 4B
No outliers; n=5 for each group.
Statistical analysis: repeated measures one-way ANOVA with Geisser-Greenhouse-correction.
Post-hoc test: Dunnett's multiple comparisons test; all group pairs tested.

Suppl. Fig. 4C
No outliers; n=7 for each group.
Statistical analysis: repeated measures one-way ANOVA with Geisser-Greenhouse-correction.
Post-hoc test: Dunnett's multiple comparisons test; all group pairs tested.

Suppl. Fig. 4D
No outliers; n=7 for each group.
Statistical analysis: repeated measures one-way ANOVA with Geisser-Greenhouse-correction.
Post-hoc test: Dunnett's multiple comparisons test; all group pairs tested.

Suppl. Fig. 4E
No outliers; n=7 for each group.
Statistical analysis: repeated measures one-way ANOVA with Geisser-Greenhouse-correction.
Post-hoc test: Tukey's multiple comparisons test; all group pairs tested.

Fig. 4 - Statistical Analyses

Fig. 4A

No outliers; n=7 for each group, except for IL-8: n=6, due to sample handling error.
pH/BE data: data for reference only; no experimental hypothesis, therefore no statistical testing performed;
Other data sets: repeated measures one-way ANOVA with Geisser-Greenhouse-correction. Post-hoc test: Tukey's multiple comparisons test; all group pairs tested.

Fig. 4B

No outliers; depicted are independent experiments (CTRL vs. Hypothermia + Histones and CTRL vs. Acidosis + Histones), separated in the graph by dotted lines.
n=7 except for:
CTRL (Hypothermia): n=6, due to handling error of sample;
Acidosis °II + Histones (Acidosis): n=6, due to extensive in-tube clotting during incubation;
Statistical analysis (Hypothermia experiment): paired t-test.
Statistical analysis (Acidosis experiment): one-way ANOVA with Geisser-Greenhouse-correction. Post-hoc test: Tukey's multiple comparisons test; all group pairs tested.

Fig. 4C

Data for reference only; no experimental hypothesis, therefore no statistical testing performed;

Fig. 4D

No outliers; n=7 for each group.
Statistical analysis: repeated measures one-way ANOVA with Geisser-Greenhouse-correction.
Post-hoc test: Tukey's multiple comparisons test; all group pairs tested.

Fig. 4E

Data for reference only; no experimental hypothesis, therefore no statistical testing performed;
